# Finding the fish factor

**DOI:** 10.7554/eLife.48459

**Published:** 2019-06-11

**Authors:** Georg Pohnert

**Affiliations:** 1 Institute for Inorganic and Analytical ChemistryFriedrich Schiller University JenaJenaGermany; 2 Max Planck Institute for Chemical EcologyJenaGermany

**Keywords:** kairomones, diel vertical migration, zooplankton, *Daphnia magna*, *Rutilus rutilus*, *Cyprinus carpio*, Daphnia

## Abstract

The water flea *Daphnia* moves to deeper waters to avoid predators when it detects a chemical produced by fish.

**Related research article** Hahn MA, Effertz C, Bigler L, von Elert E. 2019. 5α-cyprinol sulfate, a bile salt from fish, induces diel vertical migration in *Daphnia*. *eLife* **8**:e44791. doi: 10.7554/eLife.44791

In a very simplified version of the food chain found in lakes, microalgae are eaten by water fleas called *Daphnia*, which are in turn eaten by fish. But things get complicated very quickly if observed in more detail. Algae release toxins to defend themselves, and form long chains to evade predators (Van Donk et al., 2011), while *Daphnia* can change shape or move to avoid being eaten by fish.

One way that *Daphnia* and other members of the zooplankton avoid predators is by moving to different depths of the lake depending on the time of day, a strategy known as diel vertical migration. If the surrounding water contains fish, *Daphnia* move to darker, deeper regions during the day, so that the fish cannot see them (Figure 1), and move to the upper layers of the water column – where the microalgae live – at night. If there are not many fish in the vicinity, *Daphnia* stay near the surface during the day as well (Lampert, 1989).

**Figure 1.**
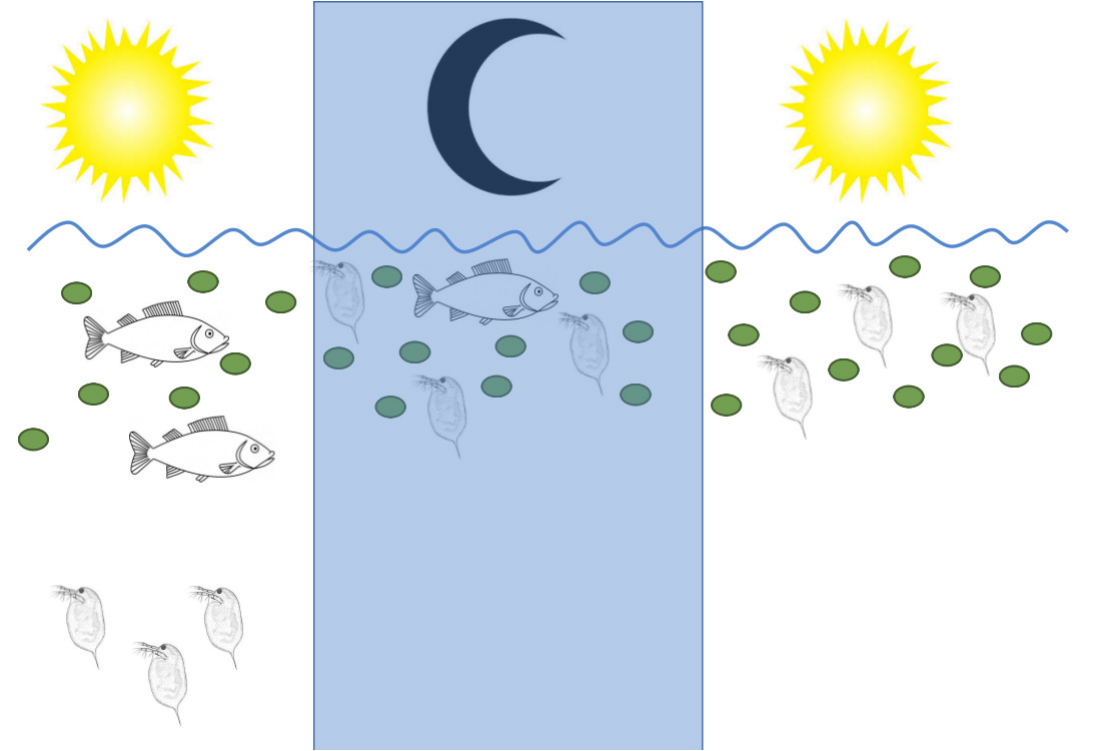
*Daphnia* water fleas change their behavior if fish are present. Left: During the day *Daphnia* migrate to deeper, darker regions of the lake if they detect chemical signals called kairomones (not shown) that are released by fish. Middle: At night, when fish cannot see them, *Daphnia* move up to the water near the surface to eat the microalgae (green circles) that are plentiful there. Right: When no fish are present, there are no kairomones to detect, and *Daphnia* stay near the surface day and night.

Prey species must balance their resources carefully. Unnecessarily avoiding predators costs energy and can restrict access to food – the microalgae eaten by *Daphnia* do not live in the dark depths of the lake – but accidentally encountering a predator can be fatal. As a result, some species have adapted to detect chemicals released by predators. The identification of several of these chemicals, called kairomones, has opened up new areas of research in aquatic ecology, conservation and aquaculture (Yasumoto et al., 2005; Selander et al., 2015; Weiss et al., 2018).

The search for the kairomone that induces diel vertical migration, also known as the ‘fish factor’, has been ongoing for decades, with spectacular failures and misinterpretations on the way (see Pohnert and von Elert, 2000 for a discussion). Numerous obstacles have complicated the search: the fish factor occurs in low concentrations in lake water, and bioassay experiments that could identify it are problematic because it is difficult to monitor the vertical movement of *Daphnia* in a laboratory setting. Now, in eLife, Meike Hahn, Christoph Effertz, Laurent Bigler and Eric von Elert report the identity of this kairomone (Hahn et al., 2019).

Hahn et al. – who are based at the University of Cologne and the University of Zurich – used a bioassay-guided fractionation method to identify the fish factor. A technique called High Performance Liquid Chromatography allowed water in which fish had previously been incubated to be separated into ‘fractions’ that each contained a subset of chemicals. Examining the effect of each fraction on the migration behavior of *Daphnia* revealed one that induced diel vertical migration even though fish were not present. Hahn et al. identified the active chemical as 5α-cyprinol sulfate. Only picomolar concentrations of this compound are found in water inhabited by fish, but even these low concentrations are sufficient to change the migration behavior of *Daphnia.*

Since the release of kairomones places predator species at a disadvantage, a prey species can only rely on them if the predator cannot shut down the production of the molecule. This is the case for 5α-cyprinol sulfate, which is a bile acid that plays an essential role in digesting dietary fats (Hofmann et al., 2010). The fish release 5α-cyprinol sulfate from their intestine, gills, and the urinary tract. As this molecule is also stable in water, it reliably indicates the presence of fish to *Daphnia*.

Besides the many implications for basic research, the finding that only picomolar amounts of a compound can trigger widespread behavioral responses in a lake also raises ecotoxicological concerns. While we survey our waters for metabolites that cause immediate toxicity, we completely ignore the fact that non-toxic doses of such highly potent signaling chemicals can also have a substantial effect on an ecosystem. This calls for a new evaluation of the routine procedures used in environmental monitoring.

Kairomones are not the only chemical signals used by the species that inhabit lakes. Pheromones (Frenkel et al., 2014), defense metabolites and molecules that help species to outcompete each other also contribute to the intricate signaling mechanisms in aquatic ecosystems (Berry et al., 2008). We can conclude that these environments are really shaped by a diverse chemical landscape, a language of life that we are only just beginning to understand.
